# Early Dynamics and Depth of Response in Multiple Myeloma Patients Treated With BCMA CAR-T Cells

**DOI:** 10.3389/fonc.2021.783703

**Published:** 2021-12-06

**Authors:** Sandy W. Wong, Nina Shah, Guy Ledergor, Thomas Martin, Jeffrey Wolf, Amy M. Shui, Chiung-Yu Huang, Joaquin Martinez-Lopez

**Affiliations:** ^1^ Bone Marrow Transplantation and Hematologic Malignancy Unit, Division of Hematology-Oncology, University of California, San Francisco, San Francisco, CA, United States; ^2^ Department of Epidemiology and Biostatistics, University of California, San Francisco, San Francisco, CA, United States; ^3^ Hospital Universitario 12 de Octubre, Complutense University, Centro Nacional de investigacion en Oncolgia (CNIO), i+12, CIBER de oncologia (CIBERONC), Madrid, Spain

**Keywords:** multiple myeloma, CAR-T, minimal residual disease, sFLC, PET-CT

## Abstract

Chimeric antigen receptor T-cell (CAR-T) therapy targeted against B-cell maturation antigen (BCMA) in multiple myeloma (MM) has produced rapid responses but many eventually relapse. In light of this new treatment, novel predictors of progression-free survival (PFS) are needed. We performed a single institution analysis of 54 BCMA-CAR-T patients. We analyzed patient’s overall response rate (ORR) by the IMWG criteria, involved serum-free light chains (iFLC), and minimal residual disease testing by next-generation sequencing (MRD-NGS). Between patients who achieved a ≤SD and those who achieved a ≥PR, PFS differed significantly (*p* < 0.0001); though there was no difference between patients who achieved a ≥CR *vs*. VGPR/PR (*p* = 0.2). In contrast, patients who achieved a nonelevated iFLC at 15 days (*p* < 0.0001, HR = 6.8; 95% CI, 2.7–17.3) or 30 days (*p* < 0.001, HR = 16.7; 95% CI, 3.9–71.7) had a prolonged PFS compared with those with an elevated iFLC. Patients achieving MRD-NGS less than the detectable limit at a sensitivity of 10^−6^ had a better PFS than those with detectable disease at 1 month (*p* = 0.02) and 3 months (*p* = 0.02). In conclusion, achieving a nonelevated iFLC and an undetectable MRD-NGS quickly were factors that were strongly associated with improved PFS. Further studies are needed to confirm the role of these markers in MM patients receiving CAR-T therapies.

## Introduction

Chimeric antigen receptor T-cell (CAR-T) therapy against B-cell maturation antigen (BCMA) is a promising new treatment for multiple myeloma (MM) with high and rapid clinical efficacy even in multiply refractory patients. By the International Myeloma Working Group (IMWG) criteria, the overall response rate (ORR) and complete response (CR) have been as high as 80%–90% and 50%, respectively (1–8). Many patients that achieve a CR are minimal residual disease (MRD) negative (1–7). However, despite high and deep responses, most patients eventually relapse (9, 10). Although the PFS is not yet mature for many constructs, bb2121 had a PFS of around 1 year (1). Manipulations of newer CAR-Ts have been focused on improving PFS. Since each novel CAR-T construct requires a long time to generate a PFS evaluation, early clinical predictors of PFS would hasten the assessment of new CAR-T improvements. Here, we report that an early assessment of involved free light chains (iFLC) and minimal residual disease (MRD) after CAR-T is closely correlated with PFS.

## Methods

We analyzed all consecutive MM patients treated with BCMA-directed CAR-T therapy from five different clinical trials (ClinicalTrials.gov: NCT03430011, NCT03361748, NCT03274219, NCT03601078, and NCT03548207) at the University of California San Francisco from November 1, 2017 to February 26, 2020. This study was approved by the local Institutional Review Board (IRB #15-17721).

All patients had serologically measurable disease prior to clinical trial entry defined as serum M-protein ≥0.5 g/dl or SFLC ≥100 mg/L. Serologic response assessments occurred at 15 days after CAR-T infusion and then monthly. Bone marrow (BM) biopsies were performed at 1, 3, 6, 12, 18, and 24 months. Plasma cell infiltration of the bone marrow core and aspirate were evaluated by conventional methods. BM clonality was defined when the κ/λ ratio is >4:1 or <1:2 for κ and λ patients by immunohistochemistry. Cytogenetics were performed on the bone marrow done before lymphodepleting chemotherapy or at screening if the patient did not receive bridging therapy. Fresh bone marrow samples from patients were sent for MRD assessment by commercially available next-generation sequencing (NGS) of immunoglobulin genes as previously described (Adaptive Biotechnologies, Seattle, WA, USA) with each BM biopsy (11–13). Imaging assessment was performed by PET-CT between 3 and 6 months after infusion. Date of data cutoff was August 27, 2020.

ORR was assessed according to the IMWG uniform response criteria (14). IMWG responses were calculated from laboratory data obtained prior to lymphodepletion chemotherapy. CAR-T toxicities such as CRS were graded using Lee criteria and ICANS was graded by CTCAEv4.03 or ICE depending on the clinical trial (15). The statistical analysis was carried out with SAS version 9.4. Hypothesis tests were two sided, and the significance threshold was set to 0.05. PFS curves were plotted using the Kaplan-Meier method, and log-rank tests were used to test for group differences. Multivariable analysis was performed by using Cox proportional hazards regression models.

## Results

Fifty-four myeloma patients were included in our analysis, with all except four having refractory disease prior to enrollment on trial (**Supplementary Table S1**). At the time of data cutoff, 26 patients relapsed, and 28 patients had ongoing responses to CAR-T treatment. Baseline characteristics were generally similar between the two groups, although the relapsed group had a higher median-involved SFLC. Both groups were heavily pretreated with a median of 6 prior lines of therapy. Overall, the median PFS was 12.7 months, similar to previously studies (1, 7). Median OS was 25.2 months. Median duration of follow-up was 8.8 months (range, 5.6–12.6).

**Figure 1:**
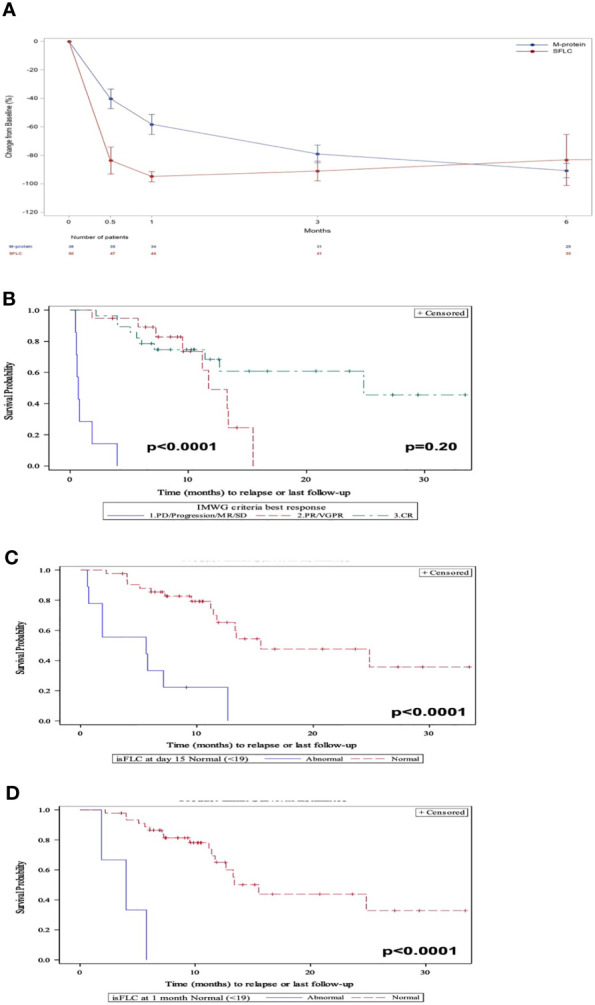
**(A)** Evolution of the M-protein and involved SFLC after CAR-T therapy in responders. **(B)** PFS by IMWG response criteria. **(C)** PFS at 15 days post-CAR-T by elevated iSFLC *vs*. nonelevated iSFLC. **(D)** PFS at 30 days post-CAR-T by elevated iSFLC *vs*. nonelevated iSFLC.

After CAR-T treatment, the involved SFLC dropped more rapidly than the M-protein in patients who responded to treatment. Over time, the depth of the M-protein decline approached that of the iSFLC (**Figure 1A**). ORR by IMWG criteria defined as ≥PR was 87% with 52% ≥CR, 35% VGPR/PR and 13% <PR. PFS differed significantly between patients who had a ≥PR compared with those who had <PR (*p* < 0.0001, HR = 63.0; 95% CI, 13.9–285.0) (**Figure 1B**). However, patients with VGPR/PR did not have a significantly different PFS from those who had a ≥CR (*p* = 0.20). Likewise, for patients with a detectable serum M-protein, its reduction was not correlated with PFS at 0.5, 1, and 3 months (*p* = 0.9, *p* = 0.8, and *p* = 0.5, respectively).

After 15 days of receiving CAR-Ts, 82% of patients had a nonelevated iSFLC which increased to 94% after 1 month. Patients who achieved a nonelevated iFLC at 15 days (**Figure 1C**) and 1 month (**Figure 1D**) had a prolonged PFS compared with those with persistently elevated iFLC (*p* < 0.0001, HR = 6.8; 95% CI, 2.7–17.3 and *p* = 0.0001, HR = 16.7; 95% CI 3.9–71.7, respectively).

**Figure 2:**
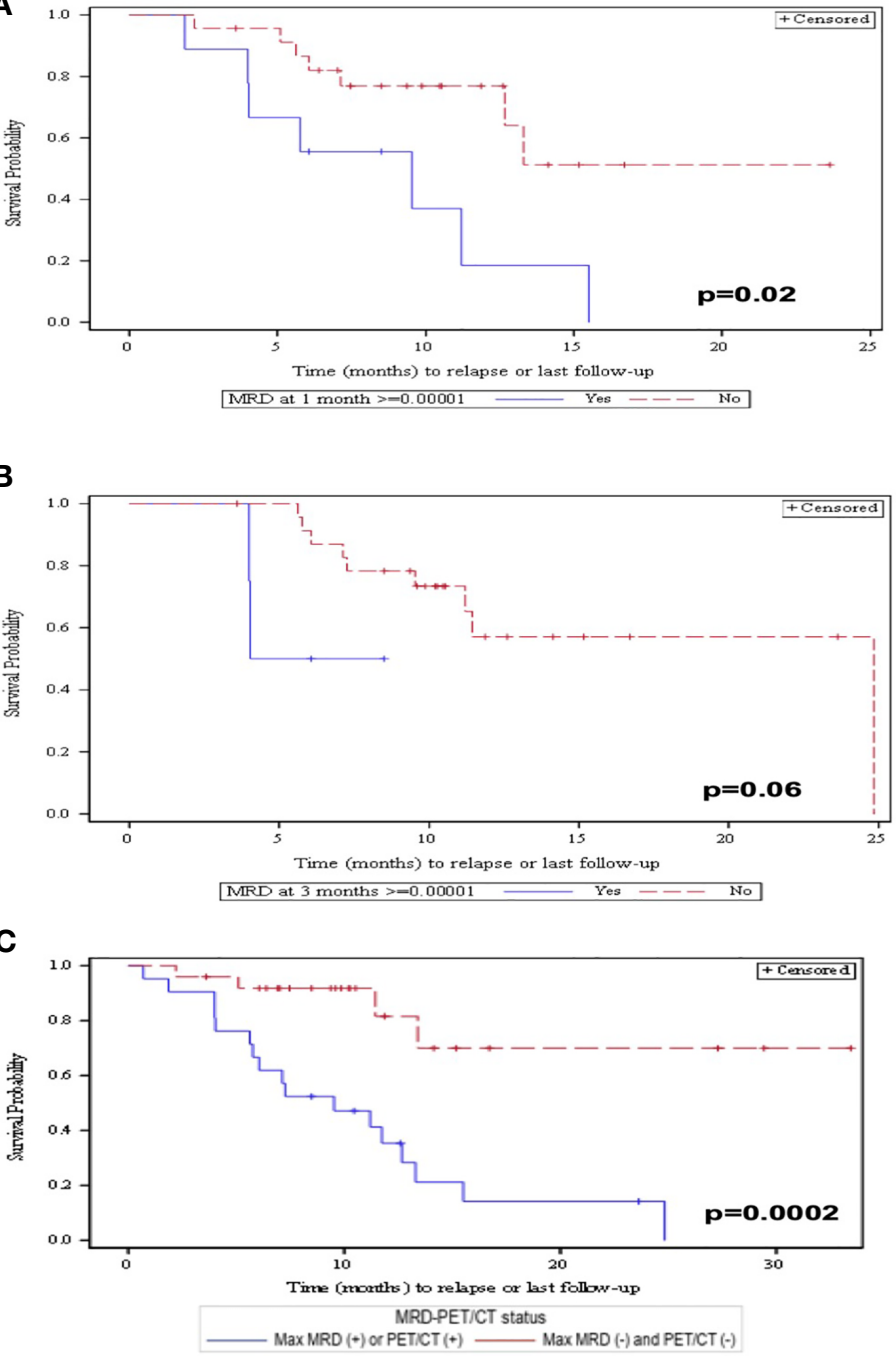
**(A)** PFS at 1 month post-CAR-T by patients who obtained MRD <10^−6^ *vs*. MRD >10^−6^. **(B)** PFS at 3 months post-CAR-T by patients who obtained MRD <10^−6^ *vs*. MRD >10^−6^. **(C)** PFS by patients who achieved MRD <10^−5^ and PET/CT negativity compared with patients who have either MRD <10^−5^ or PET/CT positivity.

Of patients who responded to CAR-T therapy, 82% achieved MRD-NGS negativity at 10^−6^ as the best response. Patients achieving MRD-NGS <10^−6^ at any point tended to have a prolonged PFS compared with those with MRD-NGS ≥10^−6^ although this did not reach statistical significance (*p* = 0.08). In contrast, patients achieving MRD-NGS ≤10^−6^ at 1 month had a prolonged PFS compared with those with MRD-NGS ≥10^−6^ (*p* = 0.02) (**Figure 2A**). Patients achieving MRD-NGS ≤10^−6^ at 3 months also had a prolonged PFS, but the association did not reach statistical significance (*p* = 0.06) (**Figure 2B**). Similar results were seen with MRD-NGS at 10^−5^. The addition of PET-CT to MRD-NGS at 10^−5^ allowed for further differentiation of patients who have a better PFS (*p* = 0.0002) (**Figure 2C**).

Lastly, 46 (85%) of the patients had CRS, nine (17%) had neurotoxicity, and 10 (19%) patients had MAS. Toxicities to CAR-T treatment such as cytokine release syndrome (CRS), neurotoxicity and macrophage activation syndrome were not associated with a better PFS (**Supplementary Figures S1SA–C**).

## Discussion

In our analysis, a ≥PR *vs*. <PR by IMWG was associated with an improved PFS but CR *vs*. VGPR/PR was not. Since most patients achieved ≥PR with CAR-T therapy, clinical and radiographic markers which correlate with better PFS are needed. Although the IMWG response criteria are the standard with which responses to myeloma treatment is graded, the criteria rely on changes in M-protein. M-protein reduction by 90% and 50% which represents the cutoff for VGPR and PR respectively, was not associated with PFS in CAR-T treated patients, neither was the M-protein reduction during the first 6 months post-CAR-T. The M-protein has a long half-life of 23 days (16). The slow clearance kinetics may limit its ability to predict PFS in the evaluation of patients whose disease has been rapidly eliminated *via* CAR-T therapy.

Instead, elevated serum free light chains at 15 days or 1-month post-CAR-T therapy were associated with worse PFS. Unlike the M-protein, SFLC have a half-life of only 2–6 h (17). Thus, the rapid clearance of SFLC may allow it to more closely mirror the myeloma burden in the body. The kinetics of M-protein and SFLC reductions with CAR-T therapy differ from studies with novel agents (18) and traditional chemotherapy (19), likely due to its unique mechanism of action.

Regarding methodology in our study, all the MRD assessments have been performed by NGS. These data should be extrapolated to MFC assessment, always that a 10^−5^ sensitivity was achieved. In the future, comparing the differences should be of interest to achieve MRD negativity between different CAR-Ts.

Our study is limited by its retrospective nature and its small sample size. Furthermore, patients in this study all had normal to near-normal kidney function, and these findings might not be generalizable to patients with renal impairment in a real-world setting, since kidney function affects light chain clearance.

In conclusion, for MM patients treated with BCMA CAR-T therapy, achieving a nonelevated iFLC as early as 15 days or 1 month, and MRD-NGS negativity with PET/CT negativity were strongly associated with a favorable PFS. Furthermore, larger studies are needed to establish the role of these markers in relation to the conventional IMWG criteria for response assessment in MM patients on CAR-T therapies.

## Data Availability Statement

The raw data supporting the conclusions of this article will be made available by the authors, without undue reservation.

## Ethics Statement

The studies involving human participants were reviewed and approved by UCSF. Written informed consent for participation was not required for this study in accordance with the national legislation and the institutional requirements.

## Author Contributions

SW, JW, and J-ML designed the study, had full access to all of the data in the study, and wrote the paper. NS, GL, TM, and JW did the data collection. AS, SW, JM-L, and C-YH performed the analysis. All authors critically revised the manuscript and gave final approval for the version to be published.

## Conflict of Interest

J-ML: speaking bureau of Adaptive and owner of shares of Altum sequencing and HOSEA. JW: consultant for Adaptive. J-ML: Honorarium and speaking bureau from BMS, Janssen, Glaxo, and Novartis. SW: Bristol-Myers Squib (research), Fortis (research), Janssen (research), Genentech (research), GSK (research), Caelum (research), Sanofi (advisory board), Telix (advisory board), Amgen (consultant), Dren (consultant).

The remaining authors declare that the research was conducted in the absence of any commercial or financial relationships that could be construed as a potential conflict of interest.

## Publisher’s Note

All claims expressed in this article are solely those of the authors and do not necessarily represent those of their affiliated organizations, or those of the publisher, the editors and the reviewers. Any product that may be evaluated in this article, or claim that may be made by its manufacturer, is not guaranteed or endorsed by the publisher.
