# Supplementary figures and images for: Early Dynamics and Depth of Response in Multiple Myeloma Patients Treated With BCMA CAR-T Cells
Source: Front Oncol. 2021 Dec 6;11:783703. doi: 10.3389/fonc.2021.783703 (PMC8685203; doi:10.3389/fonc.2021.783703)

## Slide 1
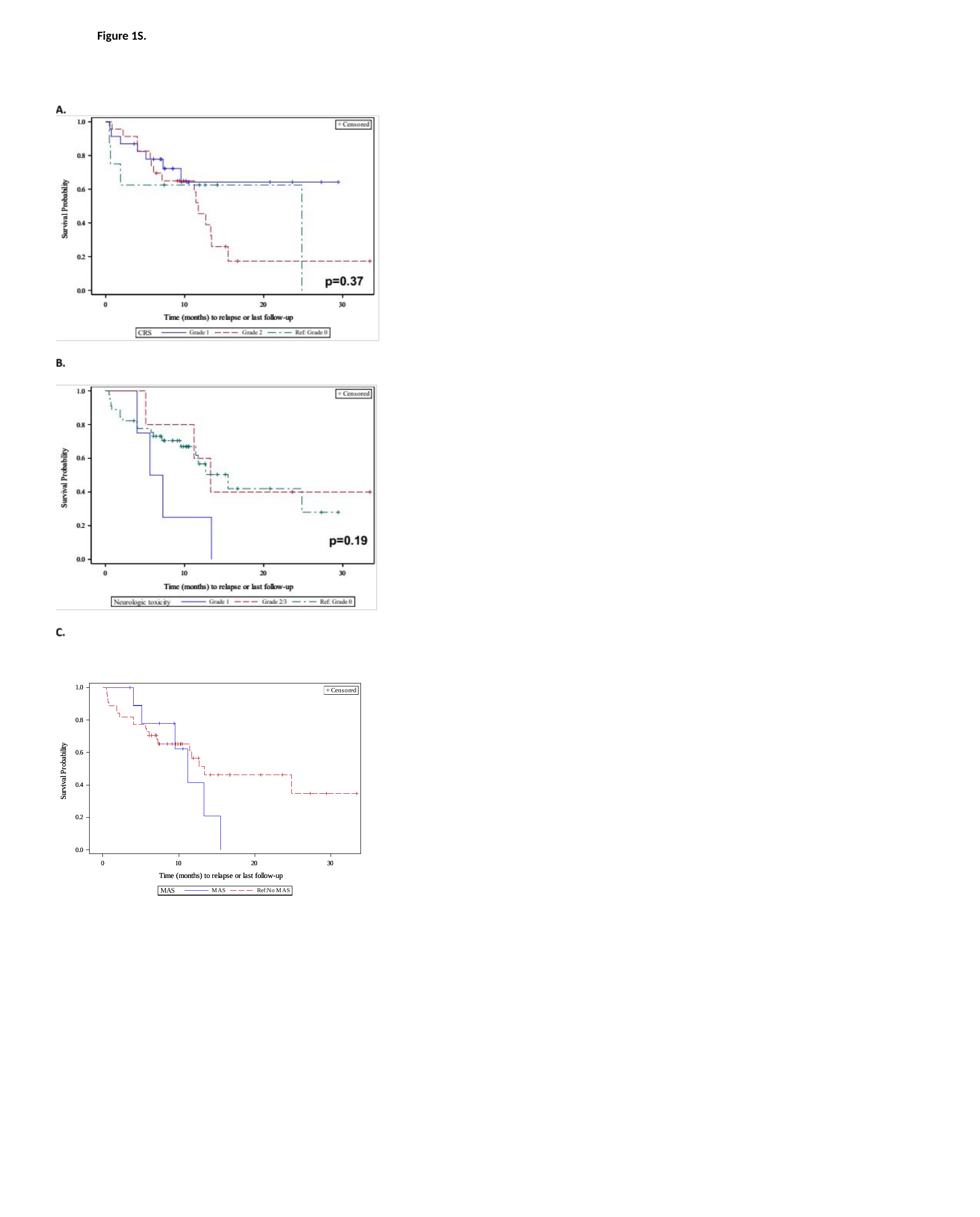

Figure 1S.

Supplement: Supplementary file 1 [file Presentation_1.pptx]
