# Supplementary material for: Early Dynamics and Depth of Response in Multiple Myeloma Patients Treated With BCMA CAR-T Cells
Source: Front Oncol. 2021 Dec 6;11:783703. doi: 10.3389/fonc.2021.783703 (PMC8685203; doi:10.3389/fonc.2021.783703)
Supplement: Supplementary file 2 [file Table_1.docx]

**Table 1S.** Baseline clinical characteristics of BMCA CAR-T treated MM patients

|  | **n = 54** | |
| --- | --- | --- |
| **Characteristic** | **Relapsed**  **(n=26)** | **Non-Relapse (n=28)** |
| Age, median, SD (range) | 61 (34-74) | 65 (43-77) |
| Female, n (%) | 13 (50) | 15 (54) |
| Myeloma type (IgG/IgA/IgD/LC), n | 16/7/0/3 | 19/5/1/3 |
| Median M-protein (g/dL) at LD, n (range) | 1.7 (0-5.2) | 1.6 (0.0-4.2) |
| Measurable Disease by M-protein, n (%) | 19 (73) | 21 (75) |
| Median involved SFLC (mg/L) at LD, n (range) | 598 (2-11054) | 65 (2-1170) |
| Median BMPC %, (range) | 0 (0-20) | 0 (0-5) |
| High-Risk Cytogenetics*, n (%) | 5 (19) | 3 (11) |
| Median prior lines of therapy, n (range) | 6 (1-13) | 5 (1-13) |
| Refractory to last line, n (%) | 26 (100) | 24 (86) |

*High-risk cytogenetics was defined as the presence of del 17p, t(4;14) or t(14;16) on pre-CAR-T therapy bone marrow biopsy.
